# TRPM4-dependent post-synaptic depolarization is essential for the induction of NMDA receptor-dependent LTP in CA1 hippocampal neurons

**DOI:** 10.1007/s00424-015-1764-7

**Published:** 2015-12-03

**Authors:** Aurélie Menigoz, Tariq Ahmed, Victor Sabanov, Koenraad Philippaert, Silvia Pinto, Sara Kerselaers, Andrei Segal, Marc Freichel, Thomas Voets, Bernd Nilius, Rudi Vennekens, Detlef Balschun

**Affiliations:** Laboratory of Ion Channel Research, Department of Cellular and Molecular Medicine, University of Leuven, Herestraat 49, bus 802, 3000 Leuven, Belgium; Laboratory of Biological Psychology, University of Leuven, Tiensestraat 102, 3000 Leuven, Belgium; TRP Research Platform Leuven (TRPLe), University of Leuven, 3000 Leuven, Belgium; Pharmakologisches Institut, Universität Heidelberg, Im Neuenheimer Feld 366, 69120 Heidelberg, Germany

**Keywords:** Transient receptor potential ion channels, Long term potentiation, Synaptic transmission, Synaptic plasticity, TRPM4

## Abstract

TRPM4 is a calcium-activated but calcium-impermeable non-selective cation (CAN) channel. Previous studies have shown that TRPM4 is an important regulator of Ca^2+^-dependent changes in membrane potential in excitable and non-excitable cell types. However, its physiological significance in neurons of the central nervous system remained unclear. Here, we report that TRPM4 proteins form a CAN channel in CA1 neurons of the hippocampus and we show that TRPM4 is an essential co-activator of *N*-methyl-d-aspartate (NMDA) receptors (NMDAR) during the induction of long-term potentiation (LTP). Disrupting the *Trpm4* gene in mice specifically eliminates NMDAR-dependent LTP, while basal synaptic transmission, short-term plasticity, and NMDAR-dependent long-term depression are unchanged. The induction of LTP in *Trpm4*^*−/−*^ neurons was rescued by facilitating NMDA receptor activation or post-synaptic membrane depolarization. Accordingly, we obtained normal LTP in *Trpm4*^*−/−*^ neurons in a pairing protocol, where post-synaptic depolarization was applied in parallel to pre-synaptic stimulation. Taken together, our data are consistent with a novel model of LTP induction in CA1 hippocampal neurons, in which TRPM4 is an essential player in a feed-forward loop that generates the post-synaptic membrane depolarization which is necessary to fully activate NMDA receptors during the induction of LTP but which is dispensable for the induction of long-term depression (LTD). These results have important implications for the understanding of the induction process of LTP and the development of nootropic medication.

## Introduction

The cellular and molecular mechanisms underlying cognitive brain functions and their deterioration by neurodegenerative and neuropsychiatric disorders are a central theme in contemporary neuroscience. It is widely accepted that during learning, complex sensorial inputs are encoded as changes in the synaptic efficacy of activated neuronal networks. At the level of individual synaptic connections, this is reflected in either long-lasting increases in synaptic efficacy (long-term potentiation (LTP)), long-lasting decreases (LTD), or a reset of previously increased or decreased efficacy to a new level (depotentiation and dedepression, respectively). Of these different forms of synaptic plasticity, LTP was the first that was discovered in the hippocampal formation [2]. Ca^2+^ influx through the *N*-methyl-d-aspartate (NMDA) subtype of glutamate receptors, upon strong post-synaptic depolarization and removal of the Mg^2+^ block from NMDA receptors (NMDAR), is widely accepted as the central trigger of LTP induction [1, 24]. Once the increase in intracellular Ca^2+^ exceeds a critical threshold value, biochemical processes necessary for LTP induction and expression are activated by molecular crosstalk within the multiprotein complex of the post-synaptic density (PSD) [20]. Many proteins and molecules have been reported to be important for LTP expression, but only a few have been identified as critical for LTP induction, such as calcium/calmodulin-dependent protein kinase II (CaMKII), cyclic adenosine monophosphate-dependent protein kinase (PKA), protein kinase C (PKC), and the extracellular signal-regulated kinase (Erk)/mitogen-activated protein kinase (MAPK) pathway [7]. In contrast to the increasing complexity of LTP mechanisms downstream of NMDAR activation, the upstream mechanisms of post-synaptic depolarization in response to pre-synaptic glutamate release are fairly established during the last decades, pointing to a dominant contribution of AMPA receptors modulated by dendritic voltage-gated Ca^2+^, Na^+^, K^+^, and I_h_ channels [3].

Here, we report a novel critical mediator of LTP induction upstream of NMDA receptor activation. We present different lines of experimental evidence, which support that activation of the transient receptor potential (TRP) channel M4 (TRPM4), a calcium-activated, but calcium-impermeable non-selective cation channel, is mandatory for NMDAR activation and the induction of LTP. The TRPM4 belongs to the melastatin subfamily of the TRP membrane proteins. TRP channels are well described for their role in sensory signaling and can be gated by a large variety of stimuli, from chemical to mechanical and to changes in temperature [11]. Among this family of 28 ion channels, TRPM4 and its closest structural relative TRPM5 exhibit some unique properties (for a review, see [23]). TRPM4 expression has been reported in a large range of tissues including several parts of the cardiovascular system and immune cells such as T cells and mast cells [17, 29]. Several studies have also detected TRPM4 messenger RNA (mRNA) and protein in the brain of rodents and humans [31, 40]. Recently, excessive TRPM4 activity has been associated with neuronal cell death in experimental autoimmune encephalomyelitis, a mouse model of multiple sclerosis [31].

## Material and methods

### Animals

*Trpm4*^*−/−*^ transgenic mice were previously described [37]. Female *Trpm4*^*−/−*^ and wild-type (WT) littermates, aged between 8 and 12 weeks, were used for all experiments. All animal experiments were in accordance with the European Community Council Directive (86/609/EC) and approved by the local ethics committee.

### In situ staining

Control and *Trpm4*^*−/−*^ mouse brains were dissected out in phosphate-buffered saline (PBS) and fixed in 4 % paraformaldehyde (PFA) for 2 h at RT and cryopreserved in 25 % sucrose overnight at 4 °C before embedding in optimal cutting temperature (OCT) compound (Tissue-Tek, Sigma-Aldrich). Sections of 12 μm were cut on a cryostat, collected on ProbeOn Plus microscope slides (Fisher Scientific), and stored at −80 °C until used.

The mouse TRPM4 (NM_175130.4) PCR product was amplified from a plasmid using the following oligonucleotides: sense 5′- **CCAGGACCGCAGTCTACCGAGTA** 3′ and antisense 5′- **GGCAAGTTAGCCCTGCGACACCT** -3′ and cloned into a pGEM easy vector (Promega). Sense and antisense digoxigenin (DIG) probes were generated in a 5-μl reaction containing 100–200 ng of linearized plasmid, using DIG RNA labeling mix (Roche Diagnostics) SP6 and T7 RNA polymerase (Invitrogen) following the manufacturer’s instructions. DIG-labeled riboprobes were ethanol-precipitated with LiCl, washed with 70 % ethanol, and resuspended in sterile DNase/RNase-free water. In situ hybridizations were performed essentially as described in [32]. Briefly, after hybridization overnight at 65 °C with a riboprobe, the slices were washed twice in 1× SSC, 50 % formamide, and 0.1 % Tween-20 at 65 °C for 30 min and blocked in the presence of 2 % blocking reagent and 20 % inactivated sheep serum. The slides were then incubated with anti-DIG-alkaline-phosphatase (AP)-conjugated antibody (Roche Diagnostics), washed, and revealed with NBT/BCIP staining.

### Protein extraction and immunoblotting

Proteins from freshly isolated hippocampus, of wild-type and *Trpm4*^*−/−*^ mice, were lysed in 1 ml of lysis buffer (100 mM Tris pH 8, 1 mM MgCl_2_, 100 μM phenylmethylsulfonyl fluoride (PMSF), and a protease inhibitors’ cocktail (10 μg/ml leupeptin and antipain, 2 μg/ml chymostatin and pepstatin)) using the Polytron homogenizer (Kinematica AG, Switzerland). After removal of nuclei, mitochondria, and any remaining large cellular fragments, pre-cleared supernatants were ultracentrifuged at 500,000 g for 30 min at 4 °C. Pellets containing total membrane fractions were solubilized, and 60 μg was subjected to SDS-polyacrylamide gel electrophoresis (SDS-PAGE) and subsequently transferred to a polyvinylidene fluoride (PVDF) membrane (Bio-Rad, USA). Respective proteins were detected with purified polyclonal rabbit anti-TRPM4 (1:1000 dilution), polyclonal rabbit anti CaMKII (1:1000, Cell Signaling), polyclonal rabbit phospho-αCaMKII (thr286) (1:1000, Cell Signaling), and monoclonal mouse anti-Na^+^/K^+^ ATPase (1: 5000 dilution) (Abcam, UK) antibodies. Immunoreactive complexes were visualized by chemiluminescence, using anti-rabbit IgG (Sigma, USA) or anti-mouse IgG (GE Healthcare) antibodies conjugated to horseradish peroxidase (1: 40,000 and 1: 5000 dilutions, respectively).

### Hematoxylin and eosin staining

Brains of control and *Trpm4*^*−/−*^ were dissected out in PBS and immersed in 4 % PFA for 2 h at RT. Samples were then placed in 25 % sucrose overnight at 4 °C for cryoprotection, embedded in OCT, and frozen. Coronal 12-μm sections containing the hippocampal area were prepared using a cryostat. Hippocampus sections were stained with hematoxylin and eosin in accordance with the standard procedure.

### Golgi staining and morphological analysis

Golgi staining was performed according to the manufacturer’s instruction (Rapid Golgi staining kit, FD Neurotechnologies, Inc., Ellicott City, MD, USA). Coronal sections (120 μm) containing identical regions of the hippocampus were selected from WT and *Trpm4*^*−/−*^ mice for analysis. Neurons chosen for analysis had to possess the following characteristics: (i) cell bodies had to be located in the middle third of the slice to avoid analysis of neurons extending into other sections; (ii) dark impregnation through all the dendrites, without breaks; and (iii) the neurons had to be isolated from the neighboring impregnated cells to avoid interferences in analysis. The branching density of the apical dendritic trees of CA1 pyramidal neurons was evaluated using the Sholl method [19], where spine density and length of dendrites arising from the soma are determined in the first- (50 μm), second- (50–100 μm), and third-order segments (100–150 μm) from the center of the soma. The number of intersections between the dendrites and the concentric circles was counted and plotted as a function of the distance from the soma. Tracing and counting were performed with a ×100 oil immersion objective (Zeiss). Curvilinear lengths were measured with Image J software. The spine density was measured on secondary branches situated in the proximal apical area (30–120 μm from the soma). On average, 4–8 neurons were included per animal, and five animals were analyzed per genotype. A MAP2 immunostaining on a separate set of slices was used to assess the neuronal density. Briefly, cryosections containing dorsal region of the hippocampus were probed with anti-MAP2 antibody (#4542; Cell Signaling) for 1 h at room temperature and revealed with anti-rabbit IgG antibody (Alexa fluor 488 conjugate; #4412; Cell Signaling). Images were acquired and analyzed using an Apotome (Zeiss). The number of pyramidal-shaped MAP2-positive cells in the CA1 area was determined per mm^2^. Three fields per slices, four slices per animal, and five animals per genotype were analyzed. All these analyses were performed blind to the genotype of subjects.

### Electrophysiology

#### Multi-electrode array recording

Mice were sacrificed by cervical dislocation, decapitated, and brains transferred to ice-cold oxygenated (95 % O_2_, 5 % CO_2_) artificial cerebrospinal fluid (aCSF; in mM): NaCl, 124; KCl, 3; NaHCO_3_, 26; NaH_2_PO_4_, 1.25; MgSO_4_, 1; glucose, 10; CaCl_2_. Hippocampi were horizontally sliced (250 μm thick) in a vibratome filled with ice-cold oxygenated aCFS. Hippocampal slices were allowed to recover in an interface chamber filled with oxygenated solution at room temperature for at least 2 h. Then, they were placed in a multi-electrode array (MEA) recording chamber and perfused continuously at a rate of 2 ml/min with aCSF at 32 °C.

Extracellular stimulations and recordings were performed with a MEA set-up (Multi Channels Systems, Reutlingen, Germany) consisting of a MEA1060-BC pre-amplifier, a filter amplifier (gain 1100×), and a two-channel stimulus generator (STG2002). MEA biochips with 8 × 8 titanium nitrite electrodes (30-μm diameter and 200-μm spacing) were used. The bath was grounded with an internal reference electrode. Slice position and contact with the electrodes were secured with a nylon mesh (ALA Scientific Instruments, USA). Stimulation electrodes were positioned at the Schaffer collateral pathway, and evoked field excitatory post-synaptic potentials (fEPSPs) were monitored at proximal stratum radiatum. Data acquisition was performed and controlled by MC_Rack 3.2.1.0 and MC_Stimulus II software (Multichannel Systems, Reutlingen, Germany).

Input/output curves were obtained 20 min after placing the slice on the biochip. Test stimuli were delivered as biphasic pulses (100-μs pulse width) at increasing voltages. For each stimulation, data from three consecutive fEPSPs were collected. Baseline stimulation strength for the rest of the experiments was set to produce 40 % of maximal response.

LTP was induced after 40 min of stable baseline by one of the following protocols: 1 or multiple trains of theta-burst stimulation (TBS, 5 bursts of 4 pulses at 100 Hz repeated at 200-ms interval) or high-frequency stimulation (HFS, 1 s at 100 Hz). LTP was expressed as the percent change in the average slope of the fEPSP taken from 40 to 60 min after LTP induction in relation to the average fEPSP slope during the 10-min baseline recording that preceded the conditioning protocol.

For electrically evoked LTD, low-frequency stimulation (LFS) consisting of 1500 pulses at 1 Hz (0.2-ms pulse width) was applied [10]. Immediately after the conditioning stimulus, evoked responses were monitored every 5 min up to 4 h. Chemically induced long-term depression [33] was induced by bath application of the selective mGluR group I agonist (S)-3,5-dihydroxyphenylglycine (DHPG; 30 μM for 15 min). In all experiments, the recording of slices from mutant mice was interleaved by experiments with wild-type controls.

Most recording also included a *paired-pulse protocol* with following interpulse intervals: 20-50-100-200 ms. Data from three consecutive fEPSPs were collected for each stimulation interval. Paired-pulse facilitation (PPF) was calculated as the ratio of the slope from the second to the first response for each paired stimulation event.

#### Whole-cell patch-clamp recording

Following decapitation, brains were isolated as described above and transverse (400 μm thick) hippocampal slices were prepared with a vibratome (MIKROM HM 650V Microm Instruments GmbH, Wallsdorf, Germany) and stored at room temperature in a holding bath containing oxygenated aCSF. After a recovery period of at least 1 h, an individual slice was transferred to the recording chamber where it was continuously superfused with oxygenated aCSF at a rate of 2.5 ml/min. Whole-cell recordings from CA1 pyramidal neurons were performed using a patch-clamp amplifier (MultiClamp 700B, Axon Instruments, Molecular Devices, Inc., Sunnyvale, CA, USA). Neuronal patching was performed under visual control by an infrared differential interference contrast optics system (Axioskop2 FC Plus, Zeiss Instruments, Jena, Germany).

To measure TRPM4 currents in CA1 pyramidal neurons from acute hippocampal slices, we performed voltage-clamp recordings during depolarizing ramps from −115 to +35 mV in 200 ms (*V*_h_ = −85 mV, *T* = 32 °C,) in the presence of high free Ca^2+^ (10 μM) in the pipette solution (132 CsMeSO_4_, 4 NaCl, 0.2 Na-GTP, 2 Mg-ATP, 2 MgCl_2_, 0.5 NaEGTA, 0.69 CaCl_2_ (10 μM free Ca^2+^), 10 K-HEPES, 5 QX-314; pH 7.27, 285 mOsm). As a control, we patched CA1 pyramidal cells from the same slices with Ca^2+^-free pipette solution (132 CsMeSO_4_, 4 NaCl, 0.2 Na-GTP, 2 Mg-ATP, 2 MgCl_2_, 2.5 NaEGTA, 10 K-HEPES, 5 QX-314; pH 7.26, 287 mOsm). Special precautions were undertaken for the sufficient dialysis of the patched cells. For this purpose, big-tip-diameter pipettes (1.5–3 MΩ) were used so that stable (80 %) Rs was always kept below 15 MΩ and recordings were started at least 15 min after establishing the w/c configuration. In order to eliminate tentative “contaminating” current components, the bath medium was supplemented with the following drugs: 2 μM TTX (voltage-gated Na^+^ channel blocker), 40 μM CNQX and 50 μM DL-AP5 (to block glutamatergic AMPA and NMDA receptors, respectively), 100 μM picrotoxin (GABA_A_ blocker), 50 μM nifedipine (L-type calcium channel blocker), 20 μM flunarizine (T-type Ca^2+^ channel blocker), and 1 μM ω-Conotoxin MVIIC (blocks N, P, and Q-type Ca^2+^ channels). Slices were perfused with this medium at least 20 min prior to recording.

To establish the basic membrane properties and intrinsic excitability of the CA1 pyramidal cells, we measured voltage responses to 500-ms duration −100 pA (hyperpolarizing) and +200 pA (depolarizing) current injections applied to the individual neuron at resting potential. Input resistance was calculated using Ohm’s law applied to the peak voltage amplitude in response to a 500-ms injection of 100-pA hyperpolarizing current. Membrane time constants were measured from the same voltage responses through single exponential fitting of the recording trace from the onset of voltage deflection to the peak of the membrane charging curve. Voltage sag (a hallmark of hyperpolarization-activated currents) was quantified as the ratio of the steady-state voltage amplitude over the peak amplitude in response to hyperpolarizing step. Action potential parameters were measured from the first spike in response to depolarizing pulse. Spike after-depolarization (ADP) was measured following a single action potential elicited by a 2-ms depolarizing current pulse of +2 nA. Current-clamp recordings were performed using borosilicate glass microelectrodes (3–5 MΩ) containing (mM) K-gluconate, 130; KCl, 20; K-Hepes, 10; EGTA, 0.2; Na-GTP, 0.3; Mg-ATP, 4; pH 7.3, 287 mOsm. The aCSF was additionally supplemented with CNQX (5 μM), AP5 (5 μM), and picrotoxin (50 μM) to block AMPA/kainate, NMDA, and GABA_A_ receptors, respectively. The liquid junction potential of 13 mV was corrected for arithmetically. Only cells with a corrected junction potential and a resting membrane potential more negative than −60 mV were used for experiments. To keep the pre-stimulus membrane potential at a set level of −60 mV, the automatic slow current injection function of the MultiClamp 700B amplifier (5-s time constant) was used.

Evoked excitatory post-synaptic potentials (eEPSPs) were induced by electrical stimulation of Schaffer collaterals through a PI-Ir electrode and recorded in current-clamp mode with the membrane potential set to −60 mV. For the measurements of the eEPSP, we used the following pipette solution (in mM): K-gluconate 135, K-HEPES 10, MgCl_2_ 3.94, Na_2_ATP 2, Na-GTP 0.2, EGTA 0.010; pH 7.27, osmolarity 282 mOsm. (Calculated with “Ca Buf” program (G. Droogmans, KU Leuven), concentration of Mg-ATP was 1.94 mM and free Mg^2+^ −2 mM.)

To determine the NMDA/AMPAR ratio, patch microelectrodes were filled with a solution containing (in mM) 135 CsMeSO_4_, 4 NaCl, 4 Mg-ATP, 0.3 Na-GTP, 0.5 EGTA, 10 K-HEPES; pH 7.24, 281 mOsm. Pipette resistance was 3–5 MΩ. Excitatory post-synaptic currents (EPSCs) were evoked with biphasic square electric pulses delivered to Schaffer collaterals. AMPA-mediated EPSCs were measured at a holding potential of −70 mV in the presence of 100 μM picrotoxin to block GABA(A) receptor-mediated inhibition. NMDA-mediated EPSCs were measured at *V*_h_ = +40 mV in the presence of CNQX (40 μM) and picrotoxin (100 μM) to block AMPA- and GABA_A_-mediated components, respectively.

In the pairing protocol, CA1 pyramidal neurons were patched with a pipette solution containing 135 mM CsMeSO_3_, 10 mM HEPES, 8 mM NaCl, 0.3 mM EGTA, 4 mM Mg-ATP, 0.3 mM Na-GTP, and 5 mM QX-314. LTP in CA1 pyramidal neurons was induced by pairing stimulation of Schaffer collaterals, for 90 s at 2 Hz, with post-synaptic depolarization at a holding potential of 0 mV in voltage-clamp mode [34]. Recordings were carried out at 25 °C. Evoked EPSC amplitudes were normalized to baseline amplitudes prior to LTP induction on a cell-by-cell basis (i.e., % of a base line mean).

### Ca^2+^ imaging

After recovery, acute hippocampal slices were incubated at room temperature for 60 min in oxygenated aCSF, containing 1 μM Fura2-AM (Biotium). Thereafter, they were placed in the recording chamber of an upright fluorescence microscope (Olympus Deutschland GmbH, Hamburg, Germany) and maintained at 37 °C using a heated (40×, water-immersion) objective and a temperature-controlled multichannel perfusion system. A concentric bipolar stimulation electrode (World Precision Instruments, Sarasota, USA) was positioned to stimulate Schaffer collateral afferent fibers. Changes in intracellular calcium were monitored in the proximal stratum radiatum (CA1 region). For excitation (at 350 and 380 nm) of Fura-2, a Polychrome V monochromator (Till Photonics, Munich, Germany) was used. A 409-nm beamsplitter and a 510/590BP emission filter allowed detection of the fluorescent signal by a high-speed EM-CMOS camera (Andor iXON+ DU885KCS-VP, Andor Technology Ltd., Belfast UK). Regions of interest were selected, and analysis was performed with Live Acquisition (LA) Software from Till Photonics (Graefelfing, Germany). Fluorescence data was acquired at 2 Hz (illumination time 200 ms at each wavelength). For offline analysis and statistics, data was exported to Origin 7 software (Originlab, Northhampton, USA). For stimulation of Schaffer collaterals, biphasic pulse trains (duration 500 ms at 100 Hz, 1-ms pulse width) were applied with an isolated pulse stimulator Model 2100 (A-M Systems, Sequim, USA) with increasing stimulation strengths of 50, 100, 200, 300, 400, and 500 μA given at a 75-s interval. Synchronization between electrical stimulation and fluorescence recording was assured via the LA software (Till Photonics, Graefelfing, Germany), which triggered both the Polychrome V monochromator and the pulse stimulator, via Patchmaster software (HEKA, Harvard Bioscience Inc., USA) and an EPC9 amplifier (HEKA, Harvard Bioscience Inc., USA). For data analysis, three active regions of interests (ROI) were selected in the proximal stratum radiatum (CA1 region). The fluorescence intensities of these were averaged. Background correction was performed, by subtracting the fluorescence level, both for 350 and 380 nm, from a nearby non-active region of the same size as the recording region, as measured in parallel. Results are presented as ∆*F* = *F* − *F*_0_. *F*_0_ represents the averaged fluorescence ratio before the start of the stimulation train. Per mouse, at least two hippocampal slices were utilized. Four mice per genotype were used for experiments.

### Chemicals

Nifedipine and isoprenaline were purchased from Sigma-Aldrich, CNQX and D-APV from Ascent Scientific (Bristol, UK), and picrotoxin and (S)-3,5-dihydroxyphenylglycine (DHPG) from Tocris Cookson (Bristol, UK).

### Statistical analysis

Data are expressed as mean ± SEM. Data were compared by two-tailed Student’s *t* test and repeated-measures ANOVA with Tukey’s post hoc analysis. Statistical analyses were performed using Graphpad Prism 5 (GraphPad Software, Inc., La Jolla, CA, USA) and SPSS 19 (Armonk, NY, USA). Statistical significance was accepted at *p* < 0.05.

## Results

### TRPM4 is localized in the pyramidal cells of the hippocampal CA1 region

**Fig. 1:**
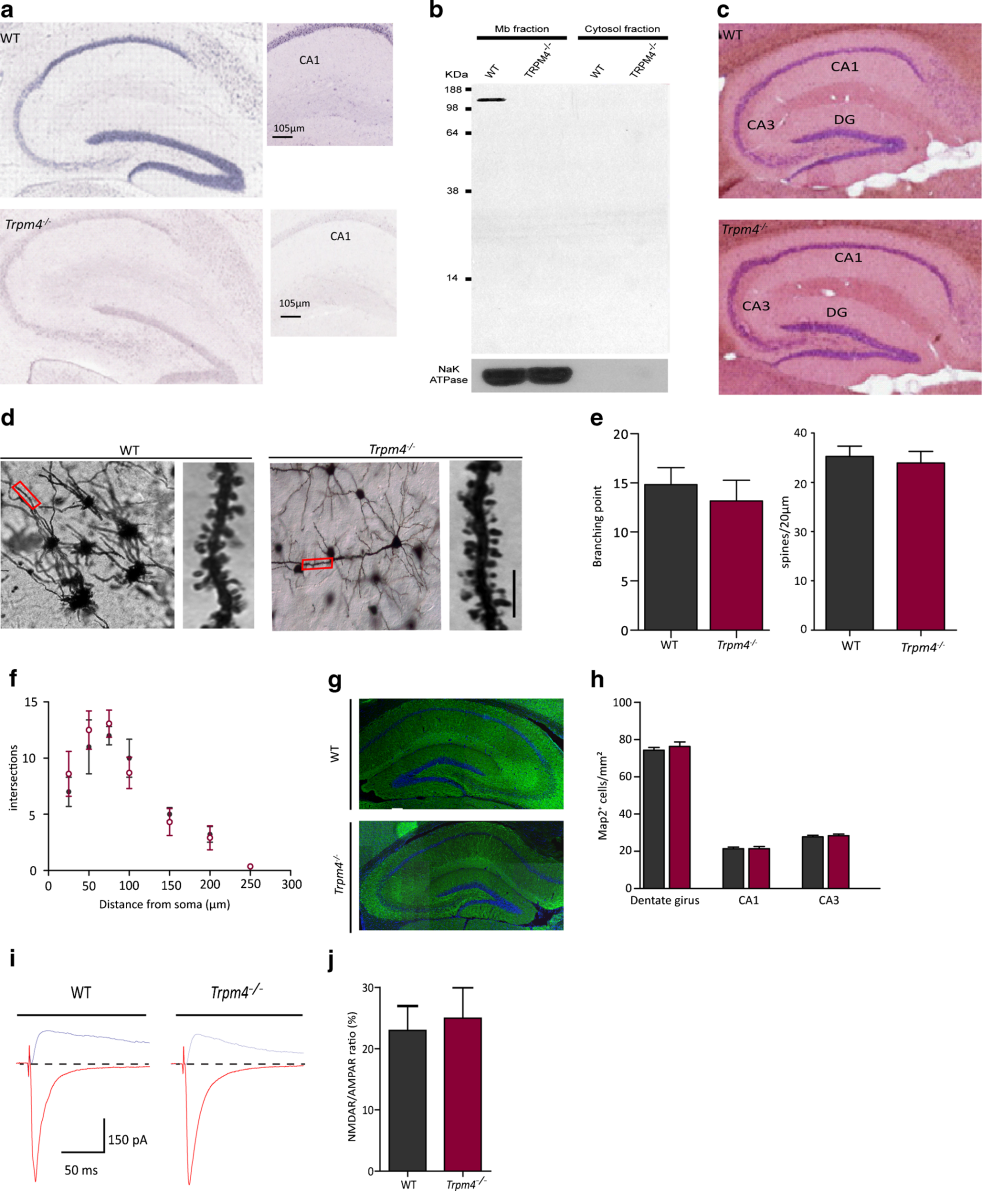
Expression pattern of TRPM4, analysis of hippocampal morphology and NMDA/AMPA ratio. **a** In situ hybridization of TRPM4 mRNA in hippocampus from WT (*upper panel*) and *Trpm4* ^*−/−*^ (*lower panel*) mice. Higher magnification pictures of the CA1 dendritic area. **b** Western blotting using TRPM4 antibody confirmed the lack of expression of TRPM4 in membrane enriched lysates (*lanes 1–2*) from hippocampi of *Trpm4* ^*−/−*^ mice as compared to WT. Note the absence of expression in cytosolic fractions (*lanes 3–4*). **c** Hematoxylin and eosin staining of hippocampus coronal cryosections (12 μm) from WT and *TRPM4* ^*−/−*^ mice. **d** Golgi staining of CA1 neurons from WT and *Trpm4* ^*−/−*^ neurons. Magnified view of apical dendritic segments from the stratum radiatum, as indicated by *red boxes*. The *scale bar* represents 5 μm. **e**, **f** Branching points, dendritic linear index (**e**), Sholl measurements (**f**) from WT and *Trpm4* ^*−/−*^ CA1 neurons, derived from Golgi staining (as in **d**). **g** Mosaic imaging of MAP2 (*green*) and DAPI (*blue*) staining on dorsal hippocampus cryosection. **h** Density of MAP2-positive cells in dorsal hippocampus. **i** Representative traces of NMDA and AMPAR currents in WT and *TRPM4* ^*−/−*^ CA1 neurons. **j** The NMDAR/AMPAR current ratio as assessed at −70 and +40 mV is not significantly changed in *Trpm4* ^*−/−*^ CA1 neurons (WT *n* = 7; *Trpm4* ^*−/−*^ *n* = 9)

We examined TRPM4 distribution in the mouse hippocampus by in situ hybridization and found TRPM4 mRNA expression in the granule cells of the dentate gyrus and pyramidal cells of the area CA1 and to a lesser extent in the CA3 pyramidal layer (Fig. 1a). Western blotting of plasma membrane-enriched lysates from hippocampus verified the presence of TRPM4 in WT but not in *Trpm4*^*−/−*^ hippocampi (Fig. 1b).

For morphological characterization, we performed Golgi staining on the hippocampus. The apical dendrites of CA1 pyramidal neurons of WT and *Trpm4*^*−/−*^ mice were not significantly different in total dendritic length and the number of branch points (Fig. 1d, e). We observed no significant differences in Sholl analysis or in spine density between the two genotypes (Fig. 1d–f).

The number of pyramidal neurons was not different between WT and *Trpm4*^*−/−*^, and the size of the cells in the regions of the hippocampus where mRNA was abundant was comparable between WT and *Trpm4*^*−/−*^, as determined with MAP2 staining (cells/mm^2^; DG WT 76.33 ± 4.16; *Trpm4*^*−/−*^ 74.33 ± 2.5; CA1 WT 21.08 ± 2.2; *Trpm4*^*−/−*^ 21.33 ± 1.53; CA3 WT 28.33 ± 3.16; *Trpm4*^*−/−*^ 27.67 ± 1.88; *N* = 5 for both genotypes; Fig. 1 g, h).

### TRPM4 deficiency does not impair basal glutamatergic neurotransmission

To study a role of TRPM4 in synaptic communication and plasticity in the hippocampus, we focused on the Schaffer collateral-CA1 synapse, whose mechanisms are particularly well explored. Given the dominant role of glutamate receptors in these processes, we first tested AMPA and NMDA receptor activity and measured evoked EPSCs (eEPSCs) from CA1 pyramidal neurons using the whole-cell voltage-clamp technique. As shown in Fig. 1i, j, AMPA receptor-mediated eEPSCs recorded at post-synaptic membrane potentials of −70 mV (in the presence of 100 μM picrotoxin) and NMDA receptor-mediated eEPSCs measured at +40 mV (in the presence of 40 μM CNQX and 100 μM picrotoxin) were identical between genotypes, excluding a shift in the ratio of NMDA to AMPA-mediated currents in *Trpm4*^*−/−*^.

**Fig. 2:**
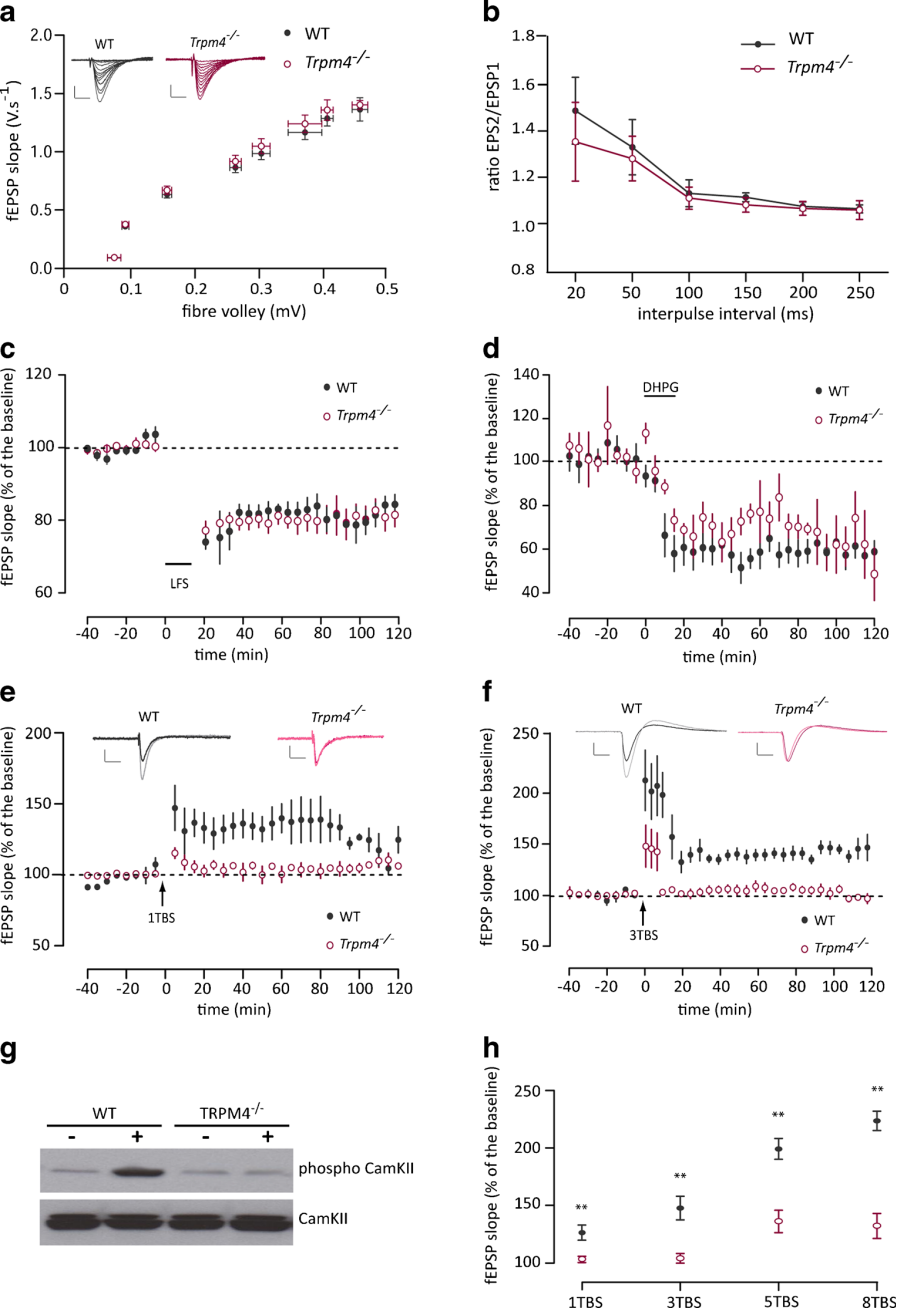
Normal basal transmission, short-term plasticity, and LTD but impaired LTP in *Trpm4* ^*−/−*^ hippocampal CA1 neurons. **a** Input/output curves of fEPSP slopes recorded from CA1 neurons at increasing stimulation intensities (0.5–4 V) did not differ between WT (*n* = 12) and *Trpm4* ^*−/−*^ (*n* = 12). Representative analog traces are shown in the *inset* (calibration 5 ms, 0.35 mV). **b** Paired-pulse facilitation (PPF) at different stimulus intervals in WT and *Trpm4* ^*−/−*^ slices. (WT *n* = 14; *Trpm4* ^*−/−*^ *n* = 12). **c** NMDAR-dependent LTD induced by low-frequency stimulation applied at time “0.” No differences in the level of depression were detected between the two groups (WT *n* = 5; *Trpm4* ^*−/−*^ *n* = 5). **d** mGluR-dependent LTD induced by bath application of the group I mGluR agonist DHPG did not differ between genotypes (WT *n* = 5; *Trpm4* ^*−/−*^ *n* = 6). **e** NMDAR-dependent LTP was induced by 1 theta-burst stimulation (TBS 5 bursts of 4 pulses at 100 Hz repeated at 200-ms interval, pulse width 100 μs) in WT (*n* = 10) but not *Trpm4* ^*−/−*^ (*n* = 10). TBS was applied to the Schaffer collateral pathway at time “0.” Representative analog traces are shown in the *inset* (calibration 5 ms, 0.35 mV). **f** Three TBS evoked NMDAR-dependent LTP only in WT (*n* = 10) while recordings of *Trpm4* ^*−/−*^ (*n* = 10) returned to baseline immediately after conditioning. Representative analog traces are shown in the *inset* (calibration 5 ms, 0.35 mV). **g** Lack of CamKII phosphorylation after a 3 TBS conditioning protocol in *Trpm4* ^-*/-*^ but not WT slices, detected by Western blotting of protein lysates from hippocampal CA1 region. **h** Changes in normalized fEPSP after different conditioning protocols (1, 3, 5, 8 TBS) are represented in function of the induction protocol (5 TBS WT, *n* = 6; TRPM4, *n* = 5, *p* = 0.0287; 8 TBS WT, *n* = 6; *TRPM4* ^*−/−*^, *n* = 5, *p* < 0.01; RM-ANOVA). Conditioning protocols were applied to the Schaffer collateral pathway at time “0”

Subsequently, we checked by multi-electrode array recordings from acute hippocampal slices of WT and *Trpm4*^*−/−*^ mice whether the absence of TRPM4 protein affects the efficacy of basal synaptic transmission. As shown in Fig. 2a, the input/output properties of field excitatory post-synaptic potentials (fEPSPs) were indistinguishable between WT and *Trpm4*^*−/−*^ slices. We also tested paired-pulse facilitation in CA1 neurons as a measure of short-term plasticity and did not detect any difference between WT and *Trpm4*^*−/−*^ at all tested interpulse intervals (Fig. 2b).

### *Trpm4*^*−/−*^ neurons exhibit deficits in NMDAR-dependent LTP

To determine whether TRPM4 plays a role in long-term synaptic plasticity, we first examined the effect of low-frequency stimulation (1 Hz) on the Schaffer collateral-CA1 synapses, which induces a NMDA receptor-dependent long-term depression (NMDAR-LTD) [10]. As shown in Fig. 2c, this conditioning protocol induced a long-lasting depression of fEPSPs that was maintained for at least 2 h, and which was indistinguishable between both genotypes (WT *n* = 5 mice; *Trpm4*^*−/−*^*n* = 5 mice; *F*_(1,8)_ = 0.53, *p* = 0.823, RM-ANOVA). Likewise, mGluR-dependent LTD induced by bath application of the specific group I mGluR agonist DHPG was not different between WT and *Trpm4*^*−/−*^ slices (WT *n* = 5 mice; *Trpm4*^*−/−*^*n* = 6 mice; *F*_(1,9)_ = 2.336, *p* = 0.161, RM-ANOVA; Fig. 2d).

Next, we induced long-term potentiation by either 1 or 3 trains of theta-burst stimulation (TBS) and examined the effect on synaptic strength. As depicted in Fig. 2e, f, the initial magnitude of potentiation was significantly lower in *Trpm4*^*−/−*^ mice. Furthermore, whereas 1 and 3 TBS induced a significant and long-lasting potentiation (>2 h) in WT, the same conditioning protocols evoked only a minimal, transient potentiation in *Trpm4*^*−/−*^ slices (1 TBS WT *n* = 10 mice; *Trpm4*^*−/−*^*n* = 10 mice; *F*_(1,18)_ = 53.869, *p* < 0.001, RM-ANOVA; 3 TBS WT *n* = 10 mice; *Trpm4*^*−/−*^*n* = 10 mice; *F*_(1,18)_ = 42.717, *p* < 0.001, RM-ANOVA).

Post-synaptically expressed LTP develops in two phases, the induction phase, which is dependent on NMDAR activation, and the expression phase, which is associated with phosphorylation and membrane insertion of AMPAR. In order to decipher whether the lack of LTP in *Trpm4*^*−/−*^ was due to a specific defect in the induction or the expression phase, we investigated the phosphorylation state of Ca^2+^/calmodulin-dependent kinase II (CamKII), which is known to be the first step of the signaling cascade upon Ca^2+^ influx through NMDA receptors [21, 35]. While the phosphorylation level of the kinase increased after TBS stimulation in WT, no difference was detectable in *Trpm4*^*−/−*^ (Fig. 2g), supporting a TRPM4-dependent defect during the induction phase, before the activation of CamKII by Ca^2+^ influx through NMDA receptors.

In the next set of experiments, we tested whether increasing the stimulation strength above the level that is commonly used for LTP induction can restore LTP in *Trpm4*^*−/−*^ slices. As shown in Fig. 2h, in response to 5 or 8 trains of TBS, significant long-term potentiation of the fEPSP was also apparent in *Trpm4*^*−/−*^. Noticeably, however, the level of potentiation was significantly lower in *Trpm4*^*−/−*^ and already saturated at 5 TBS, while in WT slices, 8 TBS still evoked more potentiation than 5 TBS (Fig. 2f; 5 TBS WT vs *Trpm4*^*−/−*^*F*_(1,9)_ = 6.763, *p* < 0.01, *n* = 6 and 5, respectively, RM-ANOVA; 8 TBS WT vs *Trpm4*^*−/−*^*F*_(1,9)_ = 16.242, *p* < 0.01, *n* = 6 and 5, respectively, RM-ANOVA).

### Voltage-dependent Ca^2+^ channel-dependent potentiation is intact in *Trpm4*^*−/−*^ CA1 neurons

**Fig. 3:**
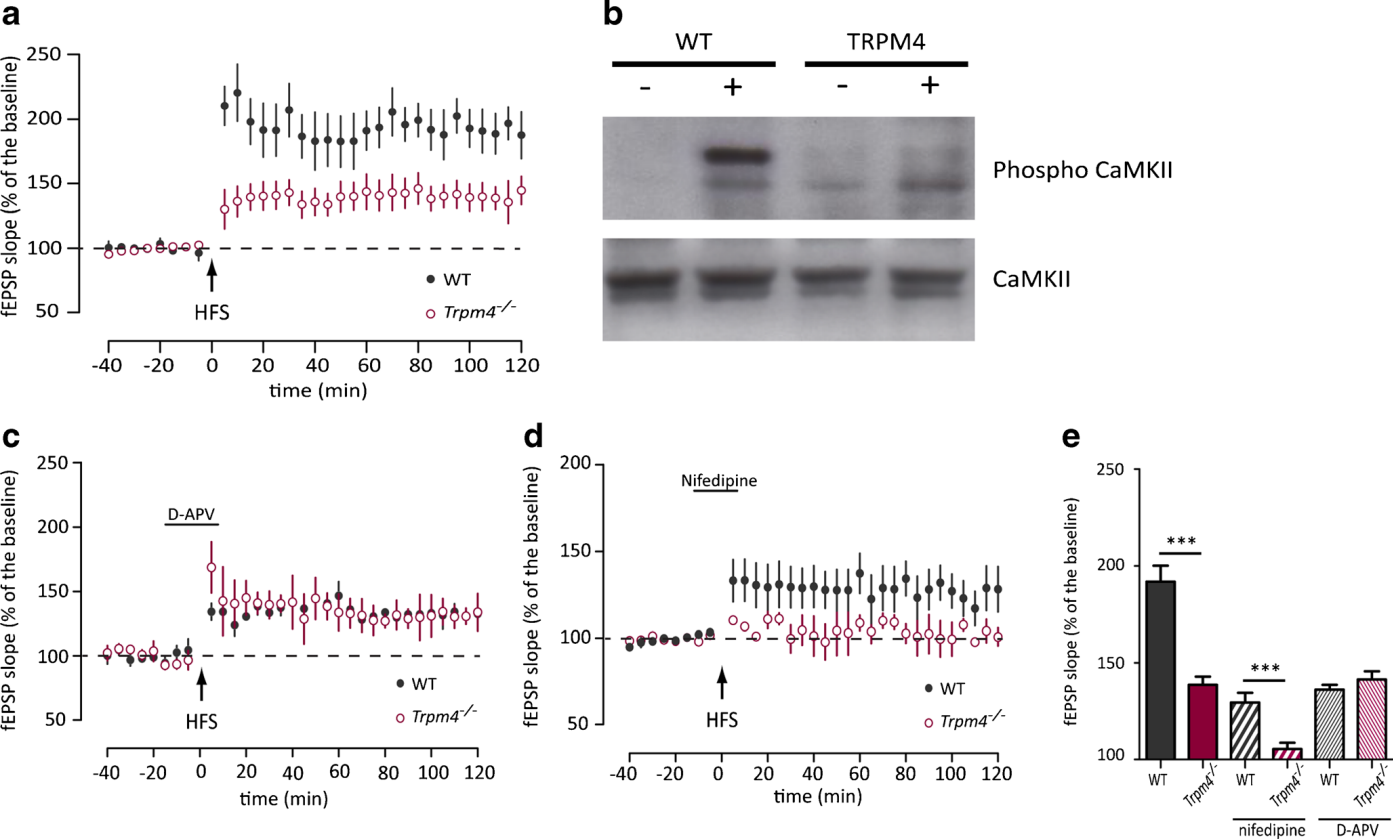
HFS-LTP is inducible through activation of VDCC in *Trpm4* ^*−/−*^ hippocampal neurons. **a** High-frequency stimulation (HFS 1-s burst at 100 Hz) of the Schaffer collateral pathway induced stronger CA1 LTP in controls (*n* = 8) as compared to *Trpm4* ^*−/−*^ slices (*n* = 7). **b** Phosphorylation of CamKII before and after conditioning protocol (3 TBS) in WT and *Trpm4* ^-/-^ slices detected by Western blotting of protein lysates from hippocampal CA1 region. **c** Application of 100 μM of the NMDAR blocker D-APV reduced HFS-induced potentiation significantly in WT (*n* = 4) but not in *Trpm4* ^*−/−*^ (*n* = 4) LTP. **d** HFS-LTP in *Trpm4* ^*−/−*^ (*n* = 5) was completely abolished by application of 100 μM of VDCC blocker nifedipine while WT still showed a significant potentiation (*n* = 5). **e** Summary of the effects of D-APV and nifedipine on LTP in *Trpm4* ^*−/−*^ and WT

Since an increasing number of TBS stimuli partially rescued the phenotype in *Trpm4*^*−/−*^ neurons, we also tested high-frequency stimulation (HFS) at 100 Hz. We used 2 trains of HFS to induce potentiation in WT and mutant slices. Although both WT and *Trpm4*^*−/−*^ slices exhibited a long-lasting potentiation of at least 2 h, the level of potentiation was significantly reduced in the *Trpm4*^*−/−*^ slices (Fig. 3a; WT *n* = 8 mice; *Trpm4*^*−/−*^*n* = 7 mice; *F*_(1,13)_ = 16.062, *p* < 0.05, RM-ANOVA). Similar as found after TBS stimulation, CaMKII phosphorylation in *Trpm4*^*−/−*^ slices was also impaired following HFS (Fig. 3b).

It has been reported that HFS induces a compound LTP, which is dependent on the activation of both the NMDARs and voltage-dependent Ca^2+^ channels (VDCC) [5, 13, 15]. To test whether the impaired potentiation in *Trpm4*^*−/−*^ could be due to a difference in activation of either of these pathways, we applied D-APV (100 μM), a specific NMDAR blocker, and nifedipine (100 μM), an L-type VDCC blocker prior to and during HFS stimulation. Application of D-APV reduced the level of potentiation in the WT while it had no effect in *Trpm4*^*−/−*^ (WT control *n* = 8 mice; APV *n* = 4 mice; *p* < 0.05 two-way ANOVA; *Trpm4*^*−/−*^ control *n* = 8 mice; APV *n* = 4 mice; n.s two-way ANOVA) (Fig. 3c). In contrast, nifedipine application totally abolished LTP in *Trpm4*^*−/−*^ and decreased potentiation in the WT group (WT control *n* = 8 mice; nifedipine *n* = 5 mice; *p* < 0.05 two-way ANOVA; *Trpm4*^*−/−*^ control *n* = 8 mice; nifedipine *n* = 5 mice; *p* < 0.05 two-way ANOVA) (Fig. 3d). These results, summarized in Fig. 3e, suggest that in *Trpm4*^*−/−*^, HFS-LTP is induced only through L-type VDCC-mediated Ca^2+^ influx, i.e., in an NMDAR-independent manner.

### Reduced excitability of *Trpm4*^*−/−*^ hippocampal CA1 neurons

**Fig. 4:**
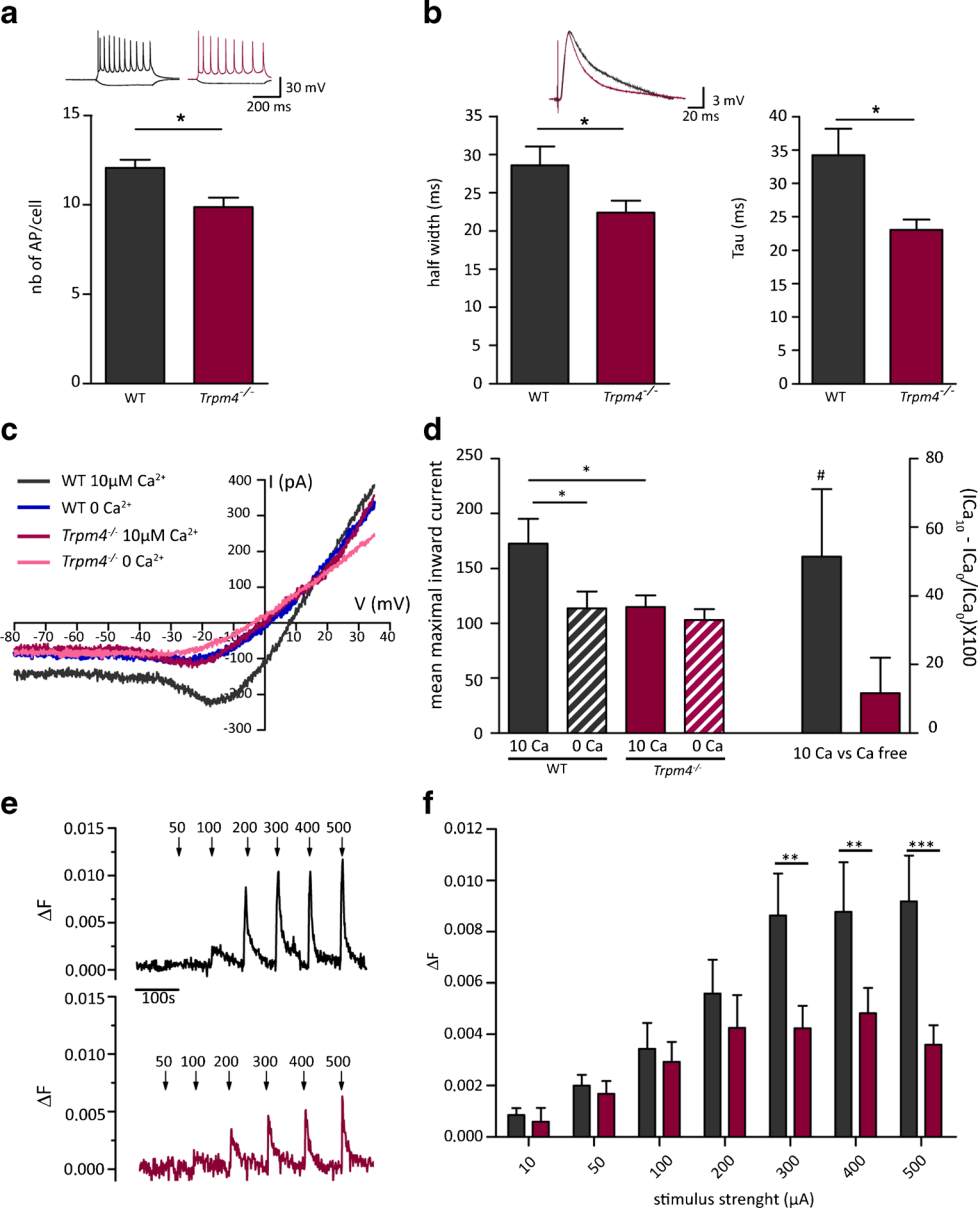
Reduced excitability of CA1 neurons in *Trpm4* ^*−/−*^ mice. **a** The number of CA1 action potentials evoked by depolarizing pulses of +200 pA for 500 ms was significantly reduced in *Trpm4* ^*−/−*^ (*n* = 6) compared to WT mice (*n* = 12) (*p* = 0.037 Student’s *t* test; representative traces are shown above). **b** Half-width and relaxation time constant of evoked EPSCs were significantly reduced in *Trpm4* ^*−/−*^ neurons (*n* = 12; WT *n* = 10, *p < 0.05). **c** Representative current traces in response to a voltage ramp from −115 to +35 mV (*V*_h_ = −85 mV). Currents are depicted in the presence of high free Ca^2+^ (10 μM) in the pipette solution or in Ca^2+^-free pipette solution, respectively. **d** *Left bars* Mean maximal inward current in CA1 hippocampal neurons from current traces as in panel **c**; *right bars* Percent increase in the TRPM4 current with 10 μM Ca^2+^ pipette solution as compared to the Ca^2+^-free condition (*Trpm4* ^*−/−*^ *n* = 12; WT *n* = 11, *p < 0.05). WT CA1 neurons show a significant current increase (#*p* = 0.0208, one-sample *t* test), in contrast to *Trpm4* ^*−/−*^ neurons (*p* = 0.2724). **e** Representative Ca^2+^ transients in the CA1 dendritic layer from WT and *Trpm4* ^*−/−*^ hippocampal slices. Stimuli (500 ms, 100 Hz) where applied at various stimulus intensities as indicated by *arrowheads* (∆*F* = *F* − *F*_0_). **f** Statistical analysis of peak Ca^2+^ signals derived from traces as in panel **e** (*Trpm4* ^*−/−*^ *n* = 12; WT *n* = 10, RM-ANOVA with Tukey’s post hoc test)

To examine the mechanism underlying the lack of LTP induction in CA1 neurons, we studied the electrophysiological properties of CA1 pyramidal cells in more detail, using whole-cell patch-clamp recordings. First, we analyzed miniature EPSPs (mEPSCs) and miniature IPSPs (mIPSCs) at Schaffer collateral CA1 synapses. We found no difference in the amplitude and frequency of mEPSCs and mIPSCs between WT and *Trpm4*^*−/−*^ mice (mEPSCs amplitude WT 11.01 ± 0.88, *n* = 6, *Trpm4*^*−/−*^ 10.67 ± 0.31 *n* = 6, *p* = 0.7268, *t* test; mEPSC frequency WT 0.67 ± 0.11 *n* = 6, *Trpm4*^*−/−*^ 0.62 ± 0.040 *n* = 6, *p* = 0.6843; mIPSC amplitude WT 17.50 ± 0.5216 *n* = 6, 16.91 ± 0.71 *n* = 6, *p* = 0.5182; mIPSC frequency WT 4.41 ± 0.89 *n* = 6, *Trpm4*^*−/−*^ 4.12 ± 0.54 *n* = 6, *p* = 0.7818). These data further support that there is no obvious defect in pre-synaptic release of exocytotic vesicles and basal synaptic transmission to CA1 principal cells. Subsequently, we compared the electrophysiological properties and stimulus-driven firing of CA1 neurons by measuring voltage responses to hyperpolarizing and depolarizing current injections (−100 and +200 pA, respectively, for 500 ms), applied at resting potential. As presented in Table 1, *Trpm4*^*−/−*^ and WT mice did not differ in electrophysiological properties of CA1 neurons, such as resting membrane potential, input resistance, action potential amplitude, and amplitude of spike after-depolarization. Notably, the number of spikes during the depolarizing step was significantly lower in *Trpm4*^*−/−*^ compared to WT mice (WT 12.3 ± 0.82, *n* = 12 mice; *Trpm4*^*−/−*^ 9.9 ± 0.79, *n* = 6 mice; *p* = 0.037 Student’s *t* test) (Fig. 4a). Since also the half-width and time constant for relaxation of evoked EPSPs were significantly reduced in *Trpm4*^*−/−*^ CA1 neurons, compared to WT neurons (Fig. 4b), this indicates that a depolarization-induced depolarizing current is reduced in *Trpm4*^*−/−*^ CA1 neurons, which contributes to the post-synaptic EPSP upon stimulation of Schaffer collaterals in WT hippocampal slices.

**Table 1:** Basal electrophysiological properties of CA1 pyramidal neurons in acute slices of *Trpm4* ^*−/−*^ and WT mice

	Wild-type	*Trpm4* ^*−/−*^
Resting *V*_m_ (mV)	−70.7 ± 1.04 (23)	−71.0 ± 1.69 (15)
Membrane time constant (ms)	25.4 ± 1.99 (21)	28.9 ± 2.54 (11)
Input resistance (MΩ)	151 ± 16 (21)	157 ± 16 (11)
AP amplitude (mV)	87.7 ± 2.01(21)	91.5 ± 2.86 (11)
AP width at 0 mV	1.07 ± 0.05 (21)	1.09 ± 0.11(11)
% sag	20.5 ± 3.3 (21)	21.2 ± 2.5 (11)
ADP amplitude (mV)	13.3 ± 0.8 (12)	14.1 ± 0.9 (7)

Mean ± SEM. *n*-Numbers are indicated in brackets and represent the number of cells. No significant differences were detected between WT and *Trpm4* ^*−/−*^

### *Trpm4*^*−/−*^ hippocampal CA1 neurons lack a Ca^2+^-dependent cation current and show diminished Ca^2+^ transients

To unveil TRPM4 channel activity in CA1 neurons directly, we performed whole-cell voltage-clamp measurements in the presence of a cocktail of blockers of glutamate receptors and voltage-gated Ca^2+^, Na^+^, and K^+^ channels. To isolate a TRPM4-dependent component from the remaining background currents, we compared currents in response to a voltage ramp from −115 to +35 mV (*V*_h_ = −85 mV), in the absence or presence of high Ca^2+^ (10 μM) in the pipette solution. Such Ca^2+^ concentration is reached in spine heads in response to depolarization [30]. In these conditions, we found in *Trpm4*^+/+^ CA1 neurons a Ca^2+^-dependent cation current with properties reminiscent of TRPM4 currents, such as *E*_rev_ around 0 mV and an outwardly rectifying *I*-*V* relation [29] (Fig. 4c). As illustrated in Fig. 4d, this current increased in WT littermates in the high-intracellular Ca^2+^ condition by 51.5 ± 19.6 % as compared to the low-intracellular Ca^2+^ condition. This Ca^2+^-dependent cation current was absent in *Trpm4*^*−/−*^ CA1 neurons, resulting in current responses in the high-intracellular Ca^2+^ condition that were not significantly different from the background currents in the low-intracellular Ca^2+^ condition (11.7 ± 10.3 %, *p* = 0.2724, one-sample *t* test; Fig. 4d, right bar graphs; Fig. 4c, d).

The above data suggest that TRPM4 contributes to post-synaptic depolarization of post-synaptic neurons upon stimulation of Schaffer collaterals, which is a critical step for full activation of NMDA receptors and initiation of LTP induction [20]. Hence, deletion of TRPM4 is expected to diminish membrane depolarization and Ca^2+^ influx via NMDA receptors, which should be detectable by Ca^2+^ imaging as smaller Ca^2+^ transients as compared to WT controls. Therefore, we measured Ca^2+^ transients in the CA1 dendritic region in response to high-frequency stimulation applied at various stimulation intensities to Schaffer collaterals. As shown in Fig. 4e, f, at low stimulation intensities, there was no difference between WT and *Trpm4*^*−/−*^ CA1 neurons. However, at high stimulation intensities, as used for LTP induction (see Fig. 3a), Ca^2+^ levels are significantly lower in *Trpm4*^*−/−*^ CA1 neurons compared to WT (Fig. 4e, f, RM-ANOVA, *p* < 0.01; *F*_(1,7)_ = 30.4).

### Rescue of impaired LTP by facilitating NMDAR activation and membrane depolarization

**Fig. 5:**
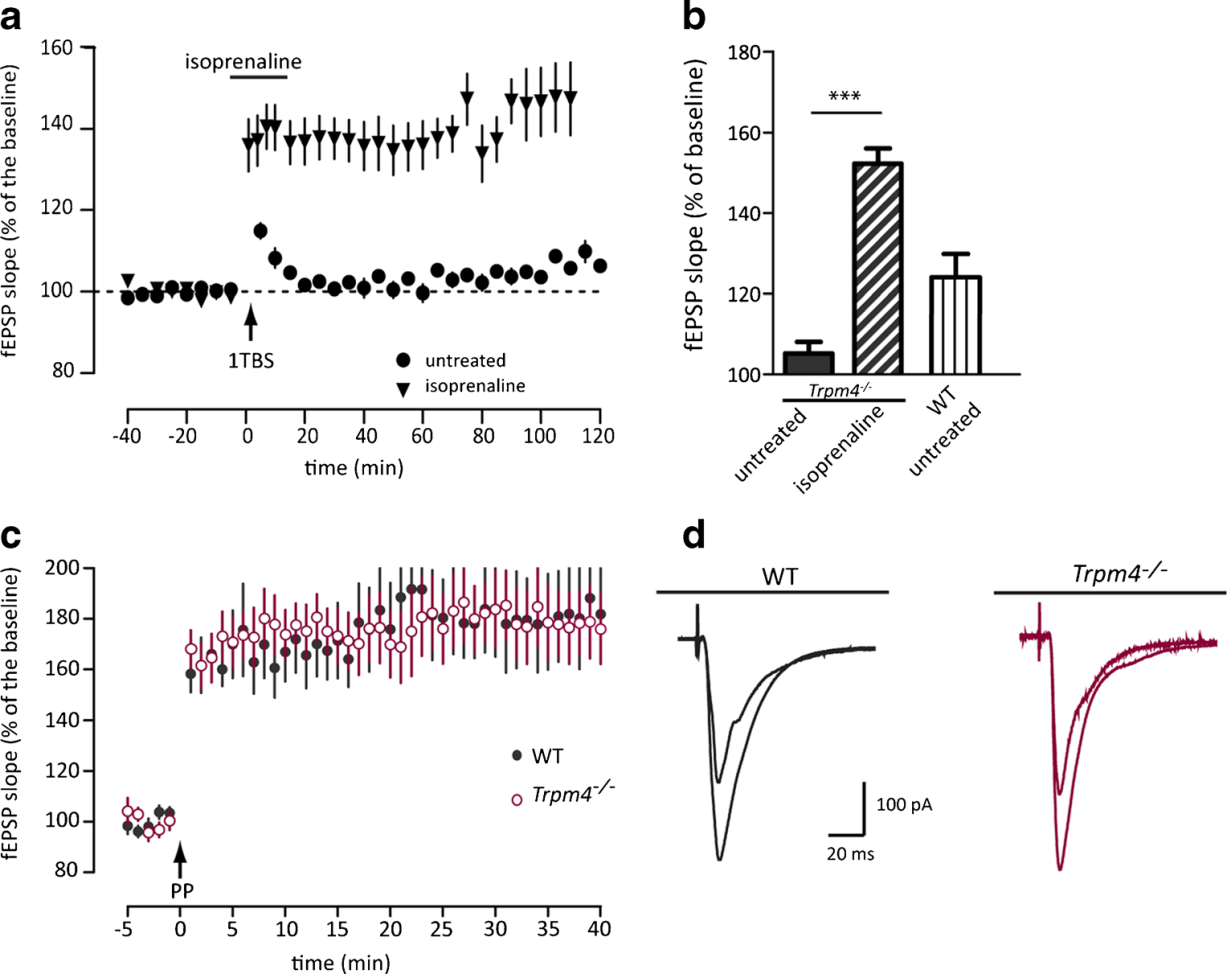
Facilitating NMDAR opening or post-synaptic depolarization rescues LTP induction in *Trpm4* ^*−/−*^ CA1 hippocampal neurons. **a** Potentiation induced with 1 TBS was rescued in *Trpm4* ^*−/−*^ (*n* = 7) by the presence 10 μM isoprenaline. **b** Comparison of mean potentiation in untreated slices and in the presence of isoprenaline during the first hour of potentiation. **c** When the same post-synaptic depolarization was imposed on *Trpm4* ^*−/−*^ and WT CA1 neurons by a pairing protocol, similar robust LTP was obtained (WT *n* = 5; *Trpm4* ^*−/−*^ *n* = 6). **d** Representative analog traces of EPSCs before and after induction of LTP of a WT and *Trpm4* ^*−/−*^ neuron, as in panel **c**

If TRPM4 activity is necessary for NMDA receptor opening, facilitation of NMDA receptor opening or applying a post-synaptic depolarization should rescue the lack of LTP induction in *Trpm4*^*−/−*^ slices. Such a rescue was achieved by increasing the excitability of *Trpm4*^*−/−*^ CA1 neurons via application of the ß-adrenergic agonist isoprenaline (10 μM; Fig. 5a, b) Previous reports described that isoprenaline rescues a lack of TBS-induced LTP in mice with a C-terminally truncated GluN2A subunit [26], likely by inhibiting the A-type K^+^ current in CA1 neurons and thus facilitating depolarization. In our hands, isoprenaline completely restored the induction of 1 TBS-LTP in *Trpm4*^*−/−*^ slices (Fig. 5a, b; *Trpm4*^*−/−*^ untreated *n* = 10 mice; *Trpm4*^*−/−*^ isoprenaline *n* = 7 mice; Bonferroni’s multiple comparison *t* = 8.322 *p* < 0.0005). In a second independent approach, we postulated that the LTP deficit should be overcome if the intrinsic depolarization is bypassed by using a pairing protocol for LTP induction under whole-cell patch-clamp conditions (similar as in [34]). This protocol imposes the same post-synaptic depolarization upon CA1 neurons of both genotypes. Indeed, as depicted in Fig. 5c, d, LTP induced by this pairing protocol was similar for both genotypes (WT *n* = 6 mice; *Trpm4*^*−/−*^*n* = 5 mice; *F*_(1,22)_ = 0.004, *p* = 0.947, RM-ANOVA). Thus, these data strongly support the hypothesis that the LTP defect is caused by a lack of sufficient post-synaptic depolarization in response to TBS or HFS stimulation.

## Discussion

In this study, we took advantage of *Trpm4* knockout mice [37] to provide the first direct evidence for a function of TRPM4, and Ca^2+^-activated non-selective cation channels in general, in neurons. Through in situ hybridization and western blotting, we show that TRPM4 is expressed in the CA1 region and the dentate gyrus of the hippocampus and to a lesser extent also in CA3 pyramidal neurons. While *Trpm4*^*−/−*^ mice are normal with regard to morphology, basal synaptic transmission, and paired-pulse facilitation at CA3-CA1 synapses, we found a complete lack of LTP induced by common theta-burst protocols to Schaffer collateral fibers. When high-intensity protocols were applied, such as 5–8 trains of theta-burst stimulation or 2 trains of high-frequency stimulation (HFS) at 100 Hz, *Trpm4*^*−/−*^ CA1 neurons developed LTP but to a lesser level than WT neurons. In contrast to LTP, NMDAR- and mGluR-dependent LTD in CA1 neurons were unchanged in *Trpm4*^*−/−*^ mice. Together, these data indicate that deletion of *Trpm4* leads specifically to an increase in the threshold for LTP induction, without affecting the requirements for induction of LTD. Patch-clamp experiments showed that AMPA and NMDA receptor currents and their ratio are indistinguishable between WT and *Trpm4*^*−/−*^ CA1 neurons, excluding changes in the activity or expression of these receptors as a reason for the observed phenotype. Instead, our data indicate an essential function of TRPM4 in the generation of a depolarizing current that is sufficiently strong to fully unblock NMDA receptors as mandatory condition for the induction of LTP. Accordingly, we have identified a Ca^2+^-dependent cation current in CA1 pyramidal neurons, which is absent in *Trpm4*^*−/−*^ mice. The lack of TRPM4 proteins leads to a reduced length of evoked excitatory post-synaptic currents and reduced excitability in CA1 neurons. Moreover, Ca^2+^ signals in CA1 neurons upon electrical stimulation of Schaffer collaterals are reduced in *Trpm4*^*−/−*^ neurons compared to WT, which is consistent with a reduction of Ca^2+^ influx due to incomplete recruitment of NMDA receptors in *Trpm4*^*−/−*^ mice. Consistent with the idea of insufficient post-synaptic depolarization as underlying cause of the observed LTP deficit, this phenotype can be completely rescued by application of isoprenaline, i.e., an intervention that facilitates the post-synaptic depolarization of CA1 neurons. Most convincingly, when the depolarization of *Trpm4*^*−/−*^ neurons is artificially clamped to the same voltage as WT neurons during a pairing protocol, LTP in both genotypes is indistinguishable with respect to amplitude and time course.

**Fig. 6:**
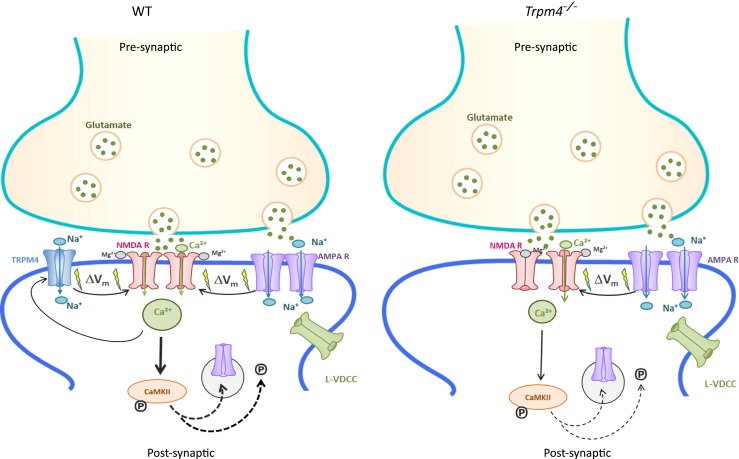
Putative function of TRPM4 in LTP induction. Pre-synaptic glutamate release causes membrane depolarization (Δ*V*_m_) via activation of AMPA receptors (AMPAR). This depolarization triggers Ca^2+^ influx through L-type voltage-dependent calcium channels (L-VDCC) and NMDA receptors (after the Mg^2+^ block at the channel pore of NMDA receptors is relieved by depolarization), depending on the conditioning protocol. The initial depolarization and the rise of [Ca^2+^]_i_ activates TRPM4 which leads to further membrane depolarization which fully unblocks the NMDAR. The resulting large intracellular calcium increase triggers phosphorylation of Ca^2+^/calmodulin-dependent kinase II (CamKII), which in turn leads to phosphorylation of inserted AMPAR and insertion of new AMPAR into the synaptic membrane. Together, this results in the induction of robust LTP. In *Trpm4* ^*−/−*^ mice, the lack of TRPM4-mediated depolarization causes only a partial removal of the magnesium block of NMDAR and a reduced calcium influx via this channel, which is insufficient to trigger LTP in *Trpm4* ^*−/−*^ mice. Facilitating NMDAR activation (with serine), reducing the inhibitory tone via the A-current (ß-adrenergic stimulation), or additional depolarization of the post-synaptic membrane in a pairing protocol rescues the induction of LTP in *Trpm4* ^*−/−*^ mice (see the text for further details)

Taken together, our data are consistent with a novel mechanistic model of LTP induction at Schaffer collateral-CA1 synapses in hippocampal slices, in which TRPM4 acts as an amplifier of the post-synaptic depolarization, which is indispensable to fully unblock the NMDA receptor and to initiate the induction of LTP in CA1 hippocampal neurons. As summarized in Fig. 6, we propose that in WT hippocampal CA1 neurons, the initial TBS stimulation leads to AMPA receptor-mediated depolarization of the post-synaptic membrane, which partially relieves the Mg^2+^ block of NMDA receptors. The resulting Ca^2+^ influx into the post-synaptic neuron activates TRPM4, which mediates a depolarizing current that further depolarizes the post-synaptic membrane, thereby fully unblocking NMDA receptors. Thus, TRPM4 activity facilitates NMDAR gating to a level necessary for activation of crucial plasticity-related downstream signal transduction cascades. In the absence of TRPM4, activation of NMDA receptors is insufficient to properly induce LTP and the downstream signaling mechanisms. In this model, it is not surprising that the reduced post-synaptic depolarization in *Trpm4*^*−/−*^ CA1 neurons is sufficient to induce NMDA receptor-dependent LTD. At resting membrane potential, the Mg^2+^ block of NMDARs is incomplete allowing still a substantial Ca^2+^ influx which is sufficient to trigger the downstream signaling pathways that mediate LTD [30]. Moreover, recent work suggested that in young animals, LTD can be induced without any Ca^2+^ influx through NMDA receptors [28].

The lack of a substantial NMDAR-mediated component of potentiation during strong stimulation and the apparent reduction to a VDCC-mediated LTP-component in *Trpm4*^*−/−*^ CA1 neurons is likely to have far-reaching consequences on Ca^2+^-dependent signaling cascades and gene activation. The ability of calcium influx to induce these events varies according to its amplitude, duration, and location [14, 30]. Whereas opening of NMDARs leads primarily to relatively long-lasting Ca^2+^ influx into dendritic spines, Ca^2+^ influx via VDCC was found to be short-lived and sensed by a multiprotein complex in the vicinity of the VDCCs that contains calmodulin and CaMKII [9, 38]. On the other hand, L-type VDCC subtypes (Ca_v_1.1. and Ca_v_1.2) bind calmodulin at carboxy-terminal recognition sites [14] enabling calmodulin to sense elevated Ca^2+^ in microdomains in the immediate vicinity [30] and to mediate the activation of signaling cascades such as the ERK/MAPK and PKA pathways, respectively [9, 39].

TRPM4 and TRPM5 are to date the only molecular candidates for the class of Ca^2+^-activated non-selective cation channels [23]. Recently, it was shown that TRPM5, but not TRPM4, contributes to slow after-depolarizations in mouse prefrontal cortex neurons [18]. Others reported that TRPM4 and TRPM5 contribute to, but are not absolutely required for, depolarization-induced slow currents in cerebellar Purkinje cells [16]. While these studies described the involvement of TRPM4 in intrinsic neuronal properties by single-cell recording, the function of TRPM4 in specific neuronal tasks and in synaptic plasticity remained unclear. Previous studies have suggested a physiological role of TRPM4 in close functional relation with glutamate receptors [8, 25, 27]. The most investigated example is the process of burst firing in the pre-Bötzinger complex neurons [8] where TRPM4-like currents have been proposed to be responsible for amplifying glutamatergic synaptic drive, by transforming the glutamatergic synaptic inputs to long-lasting membrane depolarization [25]. Mrejeru et al. have described a similar mechanism in dopaminergic (DA) neurons of substantia nigra [27]. It was shown that in those neurons, NMDA currents recruit a Ca^2+^-activated non-selective current, which can be blocked by non-specific TRPM4 blockers such as flufenamic acid and 9-phenanthrol. Using *Trpm4*^*−/−*^ mice, Schattling et al. proposed a pathophysiological role for TRPM4 in glutamate stress-induced neurodegeneration [31]. Glutamate stress is an element of the pathophysiology of experimental autoimmune encephalopathy and multiple sclerosis, which is responsible for neuronal cell death and contributes to the progressive loss of motor functions during this condition. *Trpm4*^*−/−*^ mice are partly resistant to the development of EAE, specifically because *Trpm4*^*−/−*^ neurons are resistant to cell death induced by glutamate overstimulation [31]. Our data suggest that the higher resistance of *Trpm4*^*−/−*^ neurons is likely to be caused (at least in part) by reduced Ca^2+^ influx through NMDA receptors upon glutamate stress leading to a decrease of Ca^2+^-induced neurotoxicity. Several other TRP channels are highly expressed in the brain, and some TRP channels, most notably TRPV1, have been proposed to participate in mechanisms of synaptic plasticity in the central nervous system (CNS)[36]. However, their roles in synaptic plasticity are a matter of debate (see for instance [6, 12, 22], but also [4]).

In conclusion, our data highlight TRPM4 as a key mediator for NMDAR-mediated types of LTP while it does not seem to have a function in LTD induction. Our data support a mechanism in which Ca^2+^ influx through NMDA receptors and membrane depolarization trigger the activation of TRPM4, which further amplifies membrane depolarization via a TRPM4-mediated Ca^2+^-dependent cation current in order to fully unblock NMDA receptors.
